# The hippo pathway: a molecular bridge between environmental cues and pace of life

**DOI:** 10.1186/s12862-025-02378-8

**Published:** 2025-04-24

**Authors:** Ehsan Pashay Ahi, Bineet Panda, Craig R. Primmer

**Affiliations:** 1 Organismal and Evolutionary Biology Research Programme, Faculty of Biological and Environmental Sciences, University of Helsinki, Viikinkaari 9, 00014 Helsinki, Finland; 2 Institute of Biotechnology, Helsinki Institute of Life Science (HiLIFE), University of Helsinki, Helsinki, Finland

## Abstract

The pace of life (POL) is shaped by a complex interplay between genetic and environmental factors, influencing growth, maturation, and lifespan across species. The Hippo signaling pathway, a key regulator of organ size and cellular homeostasis, has emerged as a central integrator of environmental cues that modulate POL traits. In this review, we explore how the Hippo pathway links environmental factors—such as temperature fluctuations and dietary energy availability—to molecular mechanisms governing metabolic balance, hormonal signaling, and reproductive timing. Specifically, we highlight the regulatory interactions between the Hippo pathway and metabolic sensors (AMPK, mTOR, SIRT1 and DLK1-Notch), as well as hormonal signals (IGF-1, kisspeptin, leptin, cortisol, thyroid and sex steroids), which together orchestrate key life-history traits, including growth rates, lifespan and sexual maturation, with a particular emphasis on their role in reproductive timing. Furthermore, we consider its role as a potential coordinator of POL-related molecular processes, such as telomere dynamics and epigenetic mechanisms, within a broader regulatory network. By integrating insights from molecular biology and eco-evolutionary perspectives, we propose future directions to dissect the Hippo pathway’s role in POL regulation across taxa. Understanding these interactions will provide new perspectives on how organisms adaptively adjust life-history strategies in response to environmental variability.

## Background

Pace-of-life (POL) theory is a central framework in eco-evolutionary studies, describing how organisms allocate energy and resources toward growth, reproduction, and survival in response to ecological and evolutionary pressures [1–4]. Species and individuals within populations exhibit variation in POL strategies, ranging from fast-paced life histories characterized by rapid growth, early reproduction, and short lifespans to slow-paced strategies with delayed reproduction, extended longevity, and increased investment in somatic maintenance [4, 5]. Understanding POL traits is important for deciphering how organisms adapt to environmental challenges, including resource availability and climate variability [6, 7]. These life-history strategies influence population dynamics, species interactions, and ecosystem functioning, making POL a key concept in evolutionary ecology and conservation biology [1, 7]. However, while the ecological consequences of POL variation are well recognized, the underlying molecular mechanisms that regulate these traits remain less understood, particularly how they integrate environmental signals to influence life-history trajectories [2, 8]. Among POL traits, the timing of sexual maturation is particularly significant, as it determines the onset of reproductive capacity and shapes fitness outcomes [4, 9–11]. Earlier sexual maturation is often associated with a faster POL, whereas delayed maturation aligns with a slower life-history strategy [9, 11, 12]. The timing of sexual maturation is highly plastic and sensitive to environmental conditions such as nutritional status, temperature, and stress exposure [12–15]. Shifts in reproductive timing can have profound eco-evolutionary implications, affecting population growth rates, competitive interactions, and species'resilience to environmental change. Despite its importance, the molecular regulators linking external cues to the precise control of maturation timing remain incompletely characterized, highlighting an important knowledge gap in POL research.

Several molecular mechanisms regulate POL traits, many of which also influence sexual maturation timing. Growth and metabolic pathways, such as insulin-like growth factor (IGF), mTOR, AMPK, leptin, and DLK1-Notch signaling, integrate energy availability with developmental progression [16–19]. Hormonal signals, including glucocorticoid receptor (GR), estrogen receptor (ER), and androgen receptor (AR), further regulate POL traits by mediating stress responses and reproductive axis activation [20, 21] Furthermore, senescence-related mechanisms, such as telomere length (TL) shortening, Sirtuin 1 (SIRT1), and DNA methylation, contribute to POL variation by balancing somatic maintenance and reproductive investment [22, 23]. While these pathways are well studied, how they collectively respond to environmental factors remains poorly understood. Although diet and temperature are known to influence POL traits and reproductive timing [14, 24–28], the molecular intermediaries that translate these environmental cues into biological responses remain underexplored. Identifying pathways that integrate environmental signals with POL-regulating mechanisms is important for understanding how organisms adjust life-history strategies in response to ecological pressures.

The Hippo signaling pathway, a key regulator of cell proliferation, apoptosis, and organ size [29, 30], is a strong candidate for a molecular pathway that could link POL related traits (e.g., timing of sexual maturation) to environmental stressors (e.g., dietary and thermal changes). Growing evidence suggests regulatory associations between various POL-related molecular mechanisms (e.g., telomere dynamics, growth factor, metabolic sensing and stress-related signals) and the Hippo pathway, supported by recent discoveries across various biological fields (discussed in sections below). These parallel findings highlight the need for future research to explore how these processes may intersect in shared biological roles. Notably, the links between the POL regulating mechanisms and the Hippo pathway appear particularly relevant in the context of sexual maturation and environmental factors influencing maturity (see sections 2 and 3 below). Some of the observed connections between these mechanisms and the Hippo pathway can be categorized into body size regulation, sexual maturation timing, adiposity and energy allocation, responses to thermal stress, and shared molecular interactions within these processes. For instance, telomere length (TL) is often inversely associated with body mass, suggesting that shorter TL may have co-evolved in larger, longer-lived species probably as a mechanism to suppress cancer, known as Peto's Paradox [31]. Other POL-related mechanisms such as AMPK, IGF- 1 and mTOR signals are also indicated in Peto's Paradox [32–35]. Interestingly, the Hippo pathway has been already proposed as a potential explanation for Peto's Paradox [34, 36]. The onset of sexual maturation is associated with function of these mechanisms and Hippo pathway activity not only along hypothalamo-pituitary gonadal (HPG) axis [37–40, 42, 43] but also in other tissues affecting sexual maturation such as adipose [41, 44–47]. Here, we provide an overview of recently reported associations of relevance and discuss the ecological and molecular aspects of each interaction, with the Hippo pathway playing a central role. We briefly summarize recent findings on molecular links between some of these POL-related mechanisms and the Hippo pathway, with a particular focus on the regulation of sexual maturation timing as an example of a POL-related trait. Overall, we hypothesize that the Hippo pathway can serve as a potential molecular link between POL and environmental stressors, such as dietary and thermal changes.

## Role of the Hippo pathway in sexual maturation

### Major components of the Hippo pathway

YAP (Yes-associated protein) and TAZ (transcriptional co-activator with PDZ-binding motif) are integral in controlling developmental organ size through their regulation of cellular proliferation and organ growth and initially discovered as components of the Hippo signaling pathway in *Drosophila* (Pan, 2007). As transcriptional co-activators, YAP/TAZ not only closely interact with the TEAD family of transcription factors but also engage in extensive regulatory crosstalk with other signaling pathways [30]. Within the Hippo pathway, kinase cascades like LATS1/2-MOB1 A/B and MST1/2-SAV1 are responsible for the phosphorylation of YAP/TAZ, which regulates their subcellular localization and stability [30]. This regulatory mechanism is essential for maintaining tissue homeostasis but also impacts the development and function of various organs (e.g. in reproductive tissues). SAV1 (Salvador homolog 1) and MOB1 A/B (MOB kinase activator 1 A and 1B) are scaffolding proteins that facilitate the activation of LATS1/2 by MST1/2. External stimuli, including soluble factors like epidermal growth factor family proteins and G protein-coupled receptor signals, can modulate YAP/TAZ activity via the Hippo pathway [30], highlighting the dynamic interplay between external signals and intracellular signaling mechanisms. Other transcription co-factors namely the vestigial-like (VGLL) family of co-factors, which also interact with TEADs, are key components in the Hippo signaling pathway as well [48]. In general, VGLLs compete with YAP/TAZ for binding to TEADs, thus acting as a negative regulator of YAP/TAZ-mediated transcriptional activation. Among VGLL co-factors, VGLL3 is the member with most implications in regulation of sexual maturation [48].

### Hippo pathway in sexual maturation

**Figure 1.**
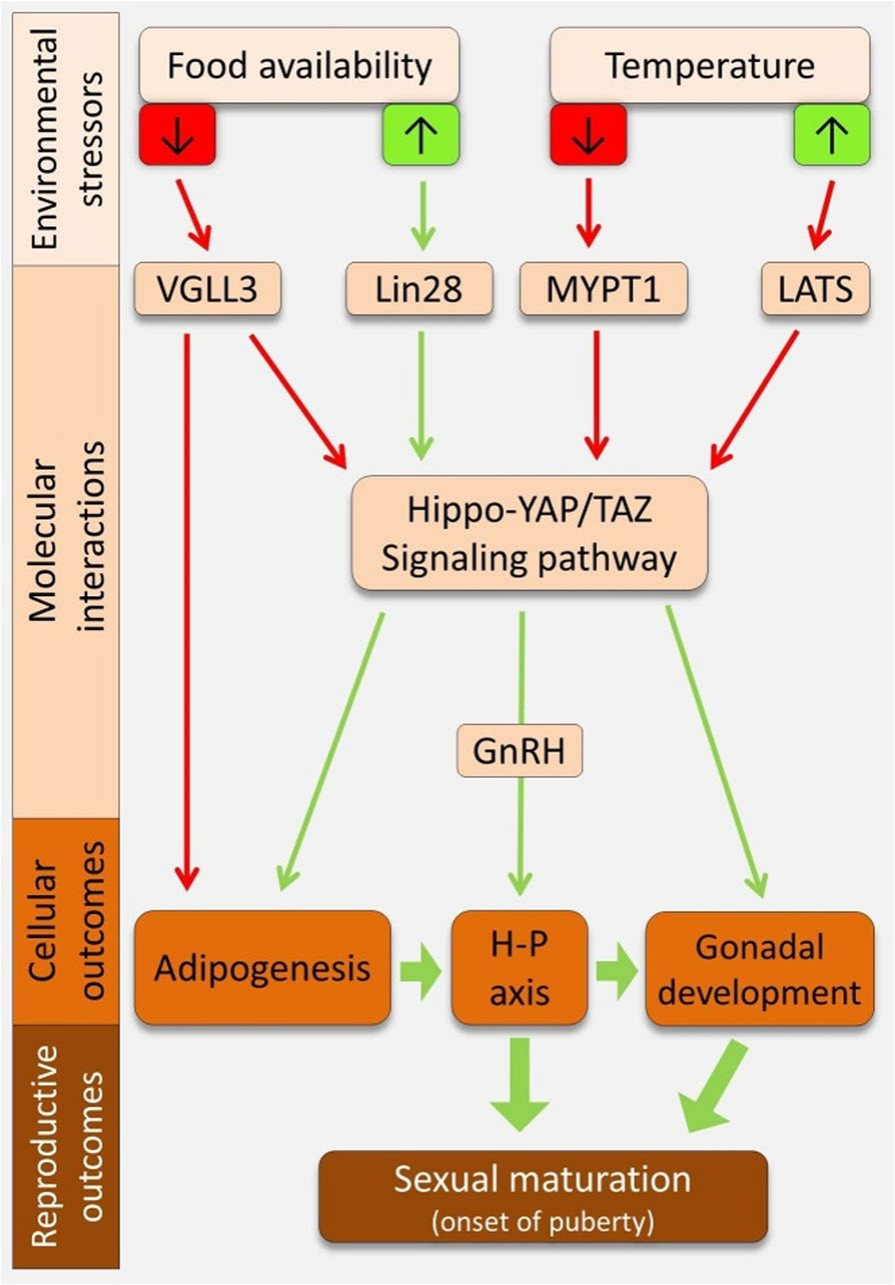
The Hippo pathway-mediated regulatory links between sexual maturation, and two related environmental stressors. To predict these regulatory connections and their downstream outcomes, the Hippo pathway is considered as a central player. The green and red arrows indicate regulatory induction and inhibition, respectively

The Hippo signaling pathway plays a key role in sexual maturation across animals [43, 49–51]. Through its downstream effectors YAP, TAZ, and VGLL co-factors (e.g., VGLL3), it influences the HPG axis, the central regulator of sexual development [43, 51, 52]. In mammals, YAP and TAZ modulate GnRH expression in the hypothalamus, affecting pituitary hormone release and gonadal function [43]. VGLL3 has been linked to pubertal timing and reproductive organ development in both mammals [53–56] and fish [39, 40, 52, 57, 58]. Beyond the HPG axis, the Hippo pathway regulates sexual maturation and reproductive capacity across species. Studies in *Drosophila* link it to germline stem cell proliferation and differentiation, essential for fertility [59], while in mammals, YAP/TAZ disruptions are associated with gonadal abnormalities [49, 51]. Other Hippo components, including MST1/2, LATS1/2 kinases, SAV1, and MOB1 A/B, further contribute to reproductive development and maturation across taxa [49] (see a summary of Hippo pathway role in sexual maturation in Figure 1).

## Hippo pathway mediated environmental effects on sexual maturation

### Hippo pathway involvement in diet-induced sexual maturation and its metabolic control

In the human population, obesity induced by a high-fat diet (HFD) is increasingly recognized as a leading cause of precocious puberty [60–62]. In female mice, postnatal feeding with HFD can even induce precocious puberty independent of body weight and body fat [63]. The HFD-induced precocious puberty in mice has been associated with changes in neural development and behaviors [64]. A recent study in rat also revealed that postnatal feeding with high-glucose diet (HGD) and HFD can both lead to precocious puberty [65].

Over the past decade, the crucial role of the Hippo signaling pathway in cellular and whole-body metabolism has emerged. Dysregulation of this pathway is linked to metabolic disorders like obesity, diabetes, and fatty liver disease. Recent studies highlight the Hippo pathway's critical role in regulating diet-induced obesity and its importance in responding to dietary changes [66]. For example, high-fat diet (HFD) induced obesity in mice found to be YAP/TAZ dependent [67]. Furthermore, HFD-induced obesity in mice causes hypertrophic adipocytes and this process has been recently found to be dependent on YAP/TAZ activation as well [68]. In human, obesity in young adults has been linked to transcriptional changes in upstream regulators of the Hippo pathway in adipose tissue. A low-calorie diet can enhance adipogenic capability by modulating these Hippo components [69]. The Hippo pathway is also recognized as a regulator of adipocyte behavior [70]. For instance, it was found that adipocytes lacking *Lats1/2* reverted to a progenitor state and while their adipocyte characteristics were reduced, their tissue remodeling abilities were enhanced [70]. In mammals, YAP mediated Hippo signaling is considered as stimulator of terminal stage of adipocyte differentiation [71], whereas *Vgll3* has been already known as a potent inhibitor of terminal stage of adipocyte differentiation [72]. *Vgll3* also regulates lipid droplet storage in the adipocytes [73]. In obese mice, disrupting Yap led to inefficient fatty acid oxidation and lipid-related toxicity, whereas, augmenting Yap levels boosted energy expenditure and reduced adiposity in adult skeletal muscle [74]. In Atlantic salmon, *vgll3*-dependent changes are also observed for adipose tissue transcriptional profile of Hippo pathway components and muscle lipid profiles [47, 75].

The role of Vgll3 in pubertal timing is conserved between humans and fish [76, 77], with vgll3 explaining over 39% of age-at-maturity variation in Atlantic salmon [78, 79]. In this species, its early maturation allele induces HPG axis genes before gonadal maturation [39, 40]. In humans, *VGLL3* is one of > 100 genes that explain 2.7% of the variation in ache at menarche [77], indicating a conserved role, but a much smaller effect size. In mice, HFD-induced precocious puberty upregulates *Lin28*, a key regulator of *GnRH* expression and energy balance [80–82]. *Lin28* inhibits the Hippo pathway while activating YAP, which in turn induces *Lin28* transcription, creating a feedback loop [83–85]. *Lin28*'s role in HPG axis regulation is conserved across vertebrates, including mice, humans, and fish [86–88]. These findings suggest the Hippo pathway acts as a conserved molecular link between diet-related metabolic changes and sexual maturation (Figure 1).

### Hippo pathway involvement in thermal control of sexual maturation

The direct evidence linking thermal regulation of age at maturity in mammals is limited, likely due to the fact that thermal effects are less important in endotherms, which regulate their body temperature internally, reducing the direct influence of external temperature on physiological and developmental processes. However, several studies of various mammalian species have shown that exposure to different temperature levels can affect the onset of sexual maturation through changes in mechanisms involving the development of reproductive organs [89–91], regulation of energy balance [92] and modulation of photoperiodic responses [93]. Unlike mammals, many studies in fish have investigated the direct effects of temperature on the onset of sexual maturation [94–98]. In addition to the abovementioned mechanisms in mammals, the thermal regulation of sexual maturation in fish can act through multiple layers of the HPG axis [97, 99]. Surprisingly though, little is still known about the detailed molecular processes mediating temperature effects on sexual maturation.

A recent study proposed an additional role for the Hippo pathway in various human cell types, whereby the effects of heat stress are mediated on the heat shock transcriptome through activation of YAP/TAZ and inhibition of LATS kinases [100]. This unexpected discovery not only revealed a previously unknown mechanism of Hippo regulation by heat stress but also demonstrated that the Hippo pathway's response to heat precedes the other already known temperature-responsive pathways [100]. Another exciting discovery found that molecular processes of cold temperature tolerance in mammals, which require complex thermoregulation in brown adipose tissue (so called beige adipogenesis), is directly dependent on YAP/TAZ co-transcriptional activity [101]. These discoveries in mammalian cells suggest an emerging critical role for the Hippo pathway in adaptation to both cold and warm environments.

Although, at the organismal level, studies demonstrating Hippo-mediated adaptation to thermal changes are very limited in mammals, such a thermal adaptive role for the Hippo pathway has been already suggested in a variety of invertebrate species [102–106]. For instance, one of the earliest studies to identify a genomic association between the Hippo pathway and adaptation to colder temperatures was conducted on honey bees (*Apis mellifera*), a species known for its high sensitivity to temperature changes [104]. Interestingly, a population genomics study revealed that small hive beetles (*Aethina tumidahas*), a parasite of bee nests, identified signals of local adaptation to various temperature gradients, with genes of the Hippo pathway also identified [102]. Further, in two congeneric oysters (*Crassostrea* spp.), adaptation to increasing temperature is a critical aquaculture trait, strong enrichment of the Hippo pathway during adaptive response to thermal stress (i.e. differential expression of genes encoding Hippo pathway components in response to increased temperature) was reported [106].

In vertebrates, several studies have implicated the Hippo pathway as being involved in mediating adaptive responses to thermal changes. For instance, in indigenous chicken breeds from different tropical climate regions (*Gallus gallus spadiceus*), genomic analyses for signatures of selection and genes involved in adaptation to high temperature identified upstream regulators of the Hippo pathway among the main candidates [107]. In giant pandas (*Ailuropoda melanoleuca*), a study identified a possible relationship between polymorphism in genes encoding Hippo pathway components and reduced inner organ sizes in the giant panda [108]. Also in pig (*Sus scrofa*), differential regulation of the Hippo pathway components has been associated with gene expression response to cold temperature in skeletal muscle; which is an important thermogenic tissue maintaining body temperature in mammals [109] (See Figure 1 for a summary of Hippo pathway-mediated thermal effects on sexual maturation).

## Pace-of-Life mechanisms involved in sexual maturation timing

**Figure 2.**
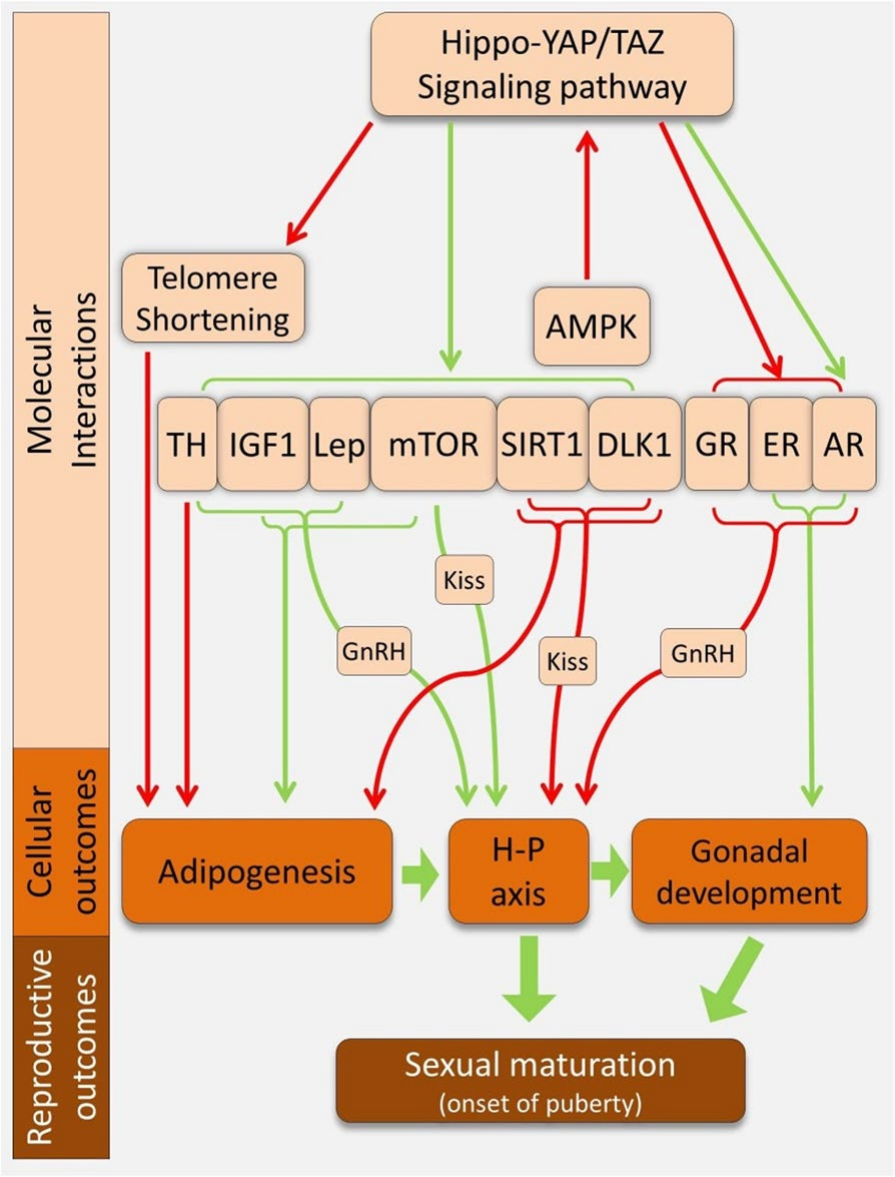
The POL-related mechanisms with regulatory connections with the timing of sexual maturation and the Hippo pathway activity. To avoid complexity, the regulatory crosstalk between mechanisms/signals is not shown; only their connections to the Hippo pathway are depicted. The green and red arrows indicate regulatory induction and inhibition, respectively

The POL mechanisms selected here represent key examples of those involved in or associated with the regulation of sexual maturation timing, particularly those with identified crosstalk with the Hippo pathway (Figure 2). These mechanisms, spanning metabolic, hormonal, and senescence-related regulators, highlight the complex molecular interactions influencing reproductive timing. However, this list is not exhaustive, as it is limited to pathways with a direct regulatory connection to the Hippo pathway, where the pathway mainly acts upstream of them.

### Growth and metabolic regulation mechanisms

#### Insulin-like growth factor

Insulin-like growth factor 1 (IGF- 1) signaling plays an important role in regulating growth, metabolism, and life-history traits, making it a key component of POL strategies [110–113]. IGF- 1 is a peptide hormone primarily produced in the liver in response to growth hormone (GH) stimulation and is a central mediator of somatic growth, energy allocation, and developmental timing [11, 114]. Across species, IGF- 1 levels correlate with growth rate, body size, and lifespan, with fast-POL species typically exhibiting higher IGF- 1 activity, promoting rapid growth and early reproductive investment, whereas slow-POL species tend to have lower IGF- 1 signaling, favoring longevity and delayed reproduction [110–113]. IGF- 1 interacts with multiple metabolic and endocrine pathways, including mTOR and AMPK, to balance energy expenditure between growth and maintenance [11, 113]. Furthermore, IGF- 1 plays a direct role in regulating the timing of sexual maturation by activating the HPG axis. It enhances GnRH secretion, increases pituitary sensitivity to gonadotropins, and influences ovarian and testicular function, thereby linking nutritional status and metabolic cues to reproductive timing [115–119]. This regulatory role highlights IGF- 1 as a key molecular pathway affected by environmental conditions and regulating POL traits and the onset of sexual maturation.

#### Mammalian target of rapamycin

The mechanistic target of rapamycin (mTOR) signaling plays role in coordinating growth, metabolism, and lifespan, making it a key regulator of POL traits [120–123]. As a central nutrient and energy sensor, mTOR integrates signals from IGF- 1, AMPK, and cellular energy availability to modulate anabolic and catabolic processes [11]. Species or individuals with a fast POL strategy are likely to exhibit higher mTOR activity, facilitating rapid growth, early maturation, and increased reproductive investment, while lower mTOR activity can be associated with slower development and extended reproductive timing in species with delayed maturation and longer lifespans [120–124]. Beyond its role in cellular metabolism, mTOR can act as an upstream regulator of the HPG axis and it exerts its effects primarily through activation of kisspeptin neurons, which serve as mediators of pubertal onset and reproductive maturation [124–126]. When energy availability is high, mTOR activation enhances *Kiss1* expression, stimulating kisspeptin release, which in turn activates GnRH neurons to initiate puberty and reproductive function. Conversely, under energy-deficient conditions, reduced mTOR activity suppresses kisspeptin expression, leading to delayed sexual maturation [124–126]. By integrating nutritional and metabolic cues with reproductive axis regulation, mTOR can act as an integrator of environmental energy status and the timing of sexual maturation.

#### AMP-activated protein kinase

AMP-activated protein kinase (AMPK) serves as a fundamental energy sensor within cells, maintaining energy balance by responding to fluctuations in intracellular ATP levels [127]. When energy is scarce, AMPK activation inhibits anabolic processes and stimulates catabolic pathways to restore ATP, thereby influencing growth, metabolism, and aging processes [128]. In the context of POL strategies, AMPK modulates life-history traits by integrating metabolic status with physiological functions [11, 129]. Importantly, AMPK plays role in regulating the timing of sexual maturation through its interaction with the HPG axis [130, 131]. Under conditions of negative energy balance, such as chronic under-nutrition, AMPK activity increases in the hypothalamus, leading to the suppression of *Kiss1* gene expression in arcuate nucleus (ARC) neurons [132]. This reduction in kisspeptin production diminishes stimulation of GnRH neurons, thereby delaying puberty onset [130, 132]. Conversely, inhibition of AMPK in kisspeptin neurons has been shown to prevent the delay in puberty caused by under-nutrition, emphasizing on the role of AMPK in linking metabolic cues to reproductive maturation.

#### Leptin

An adipocyte-derived hormone, leptin, plays a central role in the regulation of energy balance and serves as a key metabolic signal in coordinating physiological processes relevant to POL variation [133, 134]. Acting as a messenger of nutritional sufficiency, leptin reflects the body’s energy stores and communicates this status to the brain, particularly the hypothalamus, to influence feeding behavior, metabolism, and developmental timing [134–136]. Across vertebrate species, leptin levels are positively correlated with fat mass, and its signaling is known to modulate life-history traits such as growth, reproductive function, and longevity [137–139]. In fast-POL individuals or species with greater energy reserves, leptin signaling tends to be elevated, supporting early growth and reproductive investment [136–138, 140]. Importantly, leptin is a key permissive factor for the activation of the HPG axis, especially during the initiation of puberty [140–142]. Experimental studies in rodents and fish, and observations in humans with congenital leptin deficiency have demonstrated that insufficient leptin impairs the onset of puberty, while leptin administration can restore reproductive function [136, 141, 143, 144]. Mechanistically, leptin stimulates of GnRH production and release and through this, leptin integrates metabolic and energetic information with neuroendocrine signals to regulate the timing of sexual maturation, making it a molecular link between energy storage and reproductive maturation [136, 142].

#### DLK1-Notch signaling

The DLK1-Notch signaling axis plays a distinctive role in developmental regulation and energy metabolism, positioning it as a relevant pathway in the context of POL strategies [145–148]. Delta-like homolog 1 (DLK1) is a non-canonical ligand of the Notch signaling pathway, widely known for its functions in cell fate determination, tissue development, and metabolic control [147]. DLK1 is expressed in multiple endocrine tissues and has been implicated in the modulation of adipogenesis, skeletal growth, and neuroendocrine function. Its expression patterns and signaling effects suggest a role in mediating life-history trade-offs between growth, maintenance, and reproduction [148–151]. In line with POL theory, DLK1 may contribute to fast-life strategies by promoting early growth and developmental progression, while also influencing energy allocation [148]. Importantly, recent studies have identified DLK1 as a regulator of pubertal timing, particularly through its action on the hypothalamic control of reproduction [151, 152]. DLK1 appears to act upstream of the kisspeptin system, with evidence indicating that loss-of-function mutations in DLK1 can lead to central precocious puberty in humans [152, 153]. This suggests that DLK1 normally functions to restrain the onset of sexual maturation, potentially by modulating kisspeptin neuron activation or Notch-related gene networks involved in reproductive timing. Thus, DLK1-Notch signaling provides a mechanistic link between developmental and metabolic pathways and the neuroendocrine control of sexual maturation [148], contributing to how organisms balance growth and reproduction in response to internal and external cues.

### Hormonal regulation mechanisms

#### Estrogen receptor

Estrogen receptor (ER) signaling plays a multifaceted role in shaping physiological traits relevant to POL variation, particularly through its influence on growth, energy balance, and reproductive development [8, 154, 155]. Estrogens act primarily via two nuclear receptors, ERα and ERβ, which function as transcription factors regulating gene expression in target tissues, including the brain, bone, adipose tissue, and reproductive organs [154, 156]. Estrogen signaling has been linked to modulation of metabolic activity, skeletal maturation, and somatic investment, making it a key hormonal axis in balancing life-history trade-offs [154]. In the context of reproductive development, ER signaling is important for the orchestration of puberty and sexual maturation [157, 158]. Animal and human studies have demonstrated that ERα, in particular, is essential for the proper activation of the HPG axis, influencing both the structural development and functional responsiveness of hypothalamic neurons involved in reproductive control [157, 159–161]. Estrogens modulate the GnRH expression and secretion and its deficiencies, whether due to genetic mutations, pharmacological blockade, or environmental disruptions, are associated with delayed or disrupted pubertal onset, showing the importance of this pathway in the timing of sexual maturation [159–162]. By integrating signals related to internal physiological status and environmental conditions, ER pathways help calibrate reproductive timing in a manner consistent with the organism’s broader POL strategy.

#### Androgen receptor

The androgen receptor (AR), a nuclear hormone receptor activated by binding to androgens such as testosterone and dihydrotestosterone, plays role in coordinating physiological processes relevant to POL dynamics, particularly those linked to growth, metabolism, and reproductive development [8, 163, 164]. AR signaling contributes to the expression of life-history traits by influencing muscle development, metabolic rate, and tissue differentiation, all of which are critical in determining the trade-offs between somatic investment and reproductive effort [8, 163, 164]. Studies across species indicate that androgen action, mediated through AR, is not only essential for the development of secondary sexual characteristics but also plays a broader regulatory role in the maturation of the HPG axis [8, 165, 166]. The AR signaling has been shown to influence the timing of puberty by modulating hypothalamic sensitivity and the expression of genes involved in reproductive axis activation [167, 168]. For example, AR is expressed in hypothalamic neurons, including those involved in the GnRH production, and can either stimulate or inhibit neuronal circuits that control reproductive hormone secretion depending on developmental stage and androgen levels [167, 169, 170]. Disruption of AR signaling, as seen in androgen insensitivity syndromes or genetic knockout models, often results in delayed or disordered pubertal progression [164, 171]. These findings underscore the role of AR as a hormonal gatekeeper that helps fine-tune the onset of sexual maturation in alignment with an organism’s energetic and developmental status, thereby contributing to variation in POL strategies.

#### Glucocorticoid receptor

Glucocorticoid receptor (GR) signaling is a key mediator of the physiological stress response and has broad implications for POL variation, particularly through its influence on energy allocation, immune function and early development [172–174]. Activated by glucocorticoids such as cortisol or corticosterone, GRs regulate the transcription of a wide array of genes involved in metabolism, inflammation, and neuroendocrine regulation. In ecological and evolutionary contexts, elevated or prolonged glucocorticoid exposure is often associated with slower POL strategies, reflecting a shift toward energy conservation, delayed reproduction, and enhanced somatic maintenance under environmental stress [173, 175–179]. The GR signaling affects the HPG axis at multiple levels, exerting inhibitory effects on reproductive development when glucocorticoid levels are high [177, 179, 180]. Studies in rodents and primates have shown that stress-induced glucocorticoids can suppress the GnRH production and delaying sexual maturation [180–182]. This suppression is thought to be an adaptive response, delaying reproductive investment until conditions are more favorable [182, 183]. Experimental models have also revealed that GRs are expressed in hypothalamic regions critical for reproductive control, further supporting their direct role in modulating pubertal timing [183–185]. Through this stress-responsive pathway, GR signaling enables organisms to fine-tune reproductive development in relation to environmental unpredictability and internal energy status, aligning the timing of maturation with broader life-history priorities.

#### Thyroid hormone

Thyroid hormone (TH) signaling plays an essential role in regulating metabolic rate, growth, and developmental processes, positioning it as a key contributor to variation in POL strategies [186–188]. Thyroid hormones, primarily triiodothyronine (T3) and thyroxine (T4), exert their effects through nuclear thyroid hormone receptors (TRα and TRβ), which influence gene expression in a wide range of tissues, including the brain, liver, bone, and gonads [189]. Across vertebrates, TH levels have been shown to correlate with metabolic intensity and growth velocity; traits closely tied to POL variation [187, 190]. For instance, species or individuals with elevated thyroid activity often exhibit faster development, earlier maturation, and shorter generation times, characteristic of a fast POL [187, 191–193]. In contrast, reduced TH signaling is associated with delayed growth and extended developmental periods [193]. TH is also critically involved in controlling the neuroendocrine control of reproduction. It influences hypothalamic function and contributes to the maturation of the GnRH neural network, which regulates the HPG axis [194–198]. Experimental evidence in animal models indicates that thyroid dysfunction during critical developmental windows, such as hypothyroidism, can delay the onset of puberty by impairing GnRH neuron activation [144, 195, 196]. Moreover, TH is required for proper structural and functional maturation of hypothalamic circuits involved in reproductive control [199]. These findings demonstrate TH signaling as a key metabolic and developmental integrator, aligning the pace of growth and energy use with the appropriate timing of sexual maturation in accordance with the organism's broader life-history strategy.

### Senescence-related mechanisms

#### Sirtuin 1

Sirtuin- 1 (SIRT1) is a NAD⁺-dependent deacetylase widely recognized for its role in cellular stress responses, metabolism, and aging, placing it at the intersection of longevity regulation and POL strategies [200, 201]. Functionally, SIRT1 modulates gene expression by deacetylating histones and various transcription factors, thereby influencing pathways involved in DNA repair, mitochondrial function, oxidative stress resistance, and metabolic adaptation [201, 202]. Its activity is closely linked to cellular energy status, increasing under caloric restriction or low-nutrient conditions; scenarios often associated with slower POL phenotypes characterized by extended lifespan and delayed reproduction [203–205]. In the context of reproductive timing, SIRT1 has emerged as an important regulator of the (HPG axis, particularly under energy-deficient states [205]. Studies in mice have shown that elevated hypothalamic SIRT1 activity suppresses *Kiss1* expression, thereby reducing kisspeptin signaling and downstream GnRH activity, effectively delaying pubertal onset [206]. Conversely, reduced SIRT1 signaling has been associated with earlier sexual maturation, suggesting it functions as a molecular brake that adjusts reproductive timing in response to metabolic and energetic conditions [203, 205]. Through its integration of metabolic signals, epigenetic control, and reproductive axis regulation, SIRT1 serves as a key mediator aligning energy conservation and somatic maintenance with the timing of sexual maturation.

#### Telomere length dynamics

Telomere length (TL), a marker of cellular aging and replicative history, has gained attention in the study of POL variation due to its role in balancing somatic maintenance and life-history investment [207, 208]. Telomeres, which cap and protect chromosome ends, progressively shorten with cell division and oxidative stress, ultimately limiting cellular lifespan [207–210]. Species or individuals with a fast POL tend to exhibit more rapid telomere attrition, reflecting early growth, high metabolic activity, and shorter lifespans, while slow-POL strategies are often associated with longer telomeres and enhanced cellular maintenance [37, 211–217]. Studies in humans and nine-spined sticklebacks (*Pungitius pungitius*) have reported associations between TL and the timing of sexual maturation [37, 218–220]. However, the correlations between TL and various reproduction-related timings remain associative [215–217, 221–225], and there is currently no established mechanistic pathway directly linking TL dynamics to the HPG axis. Instead, one plausible explanation lies in regulatory links between telomere shortening and adipogenesis/dietary changes; both processes tightly influencing each other [226–230]. Short telomeres are known to impair adipocyte differentiation and function, potentially affecting energy storage and metabolic signaling. Since energy reserves are critical cues for pubertal onset, especially in vertebrates, it is possible that TL influences sexual maturation indirectly, by modulating the capacity for fat accumulation and the downstream metabolic signals that inform the brain about readiness for reproduction. Thus, while telomere dynamics may reflect POL trade-offs, their role in regulating maturation timing likely operates through indirect effects on energy availability rather than direct control of reproductive signaling pathways.

## The Hippo pathway as a master regulator of POL related mechanisms

**Figure 3.**
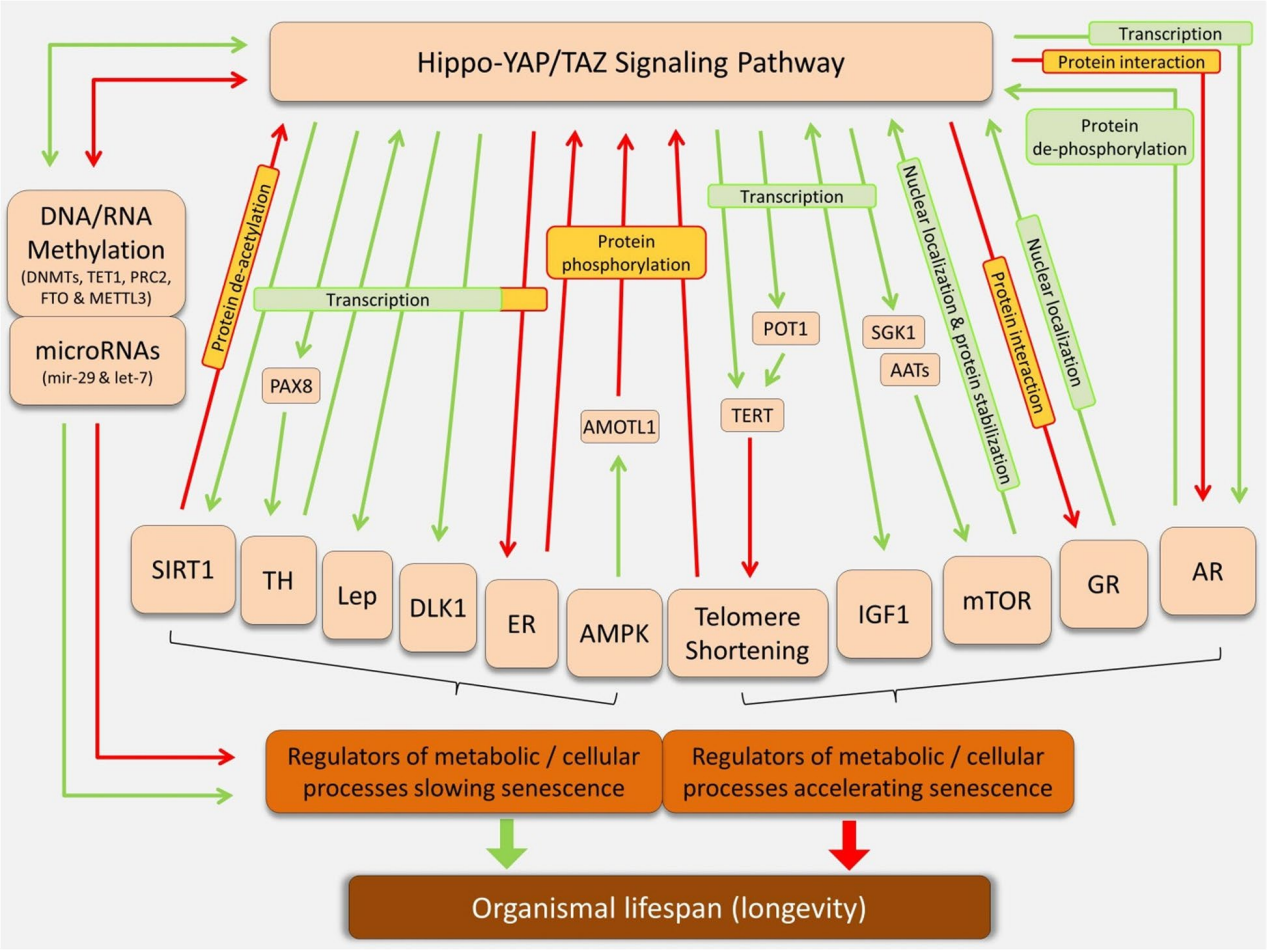
Direct regulatory connections between the Hippo pathway and POL-related mechanisms influencing organismal lifespan. To avoid complexity, the regulatory crosstalk between mechanisms/signals is not shown; only their connections to the Hippo pathway are depicted. The green and red arrows indicate regulatory induction and inhibition, respectively

The Hippo signaling pathway can be considered a master regulator of the abovementioned POL-related mechanisms, given its ability to act upstream of all of them and regulate their activity. While it is also regulated by some of these pathways, its primary role as a central coordinator places it at the heart of a broader regulatory network. Although the Hippo pathway’s involvement in specific POL traits—such as body size and sexual maturation timing—has gained increasing attention, its potential role in other life-history traits like aging, longevity, and lifespan remains largely unexplored. Nevertheless, since many Hippo-connected POL mechanisms are well-established regulators of cellular aging, it is reasonable to speculate that Hippo signaling may contribute more broadly to life-history evolution. In the following sections, we provide examples of direct regulatory connections between the Hippo pathway and the activity of the POL-related mechanisms, divided into two main groups: those associated with accelerated aging, and those linked to aging delay and extended lifespan (summarized in Figure 3).

### Hippo pathway interactions with POL mechanisms associated with accelerated aging

#### IGF- 1 signaling

While essential for growth and development, chronic elevation of IGF- 1 signaling has been associated with increased cancer risk, reduced stress resistance, and shorter lifespan in multiple species [231–233]. High IGF- 1 activity promotes anabolic growth and cell proliferation, which may increase the burden of DNA damage and reduce investment in repair mechanisms over time. Animal studies consistently show that reduced IGF- 1 signaling extends lifespan, supporting its role in accelerated aging when overly active [234, 235]. In recent years, studies in mammalian cells have revealed a direct regulatory connection between YAP and the IGF- 1 receptor at the transcriptional level, with evidence suggesting that this interaction is reciprocal and stimulatory in both directions [236–239]. However, most of these studies have been conducted under pathological conditions (excessive cell proliferation), and such regulatory connections need to be further investigated during normal tissue growth and at the organismal level.

#### mTOR signaling

mTOR integrates nutrient, energy, and growth signals to drive protein synthesis and cell growth, but its persistent activation has been closely tied to cellular aging, metabolic dysfunction, and reduced longevity [236]. Overactive mTOR signaling suppresses autophagy, contributing to the accumulation of damaged proteins and organelles, a hallmark of aging cells [237]. Pharmacological inhibition of mTOR has been shown to extend lifespan in various model organisms [238–240]. During lipogenesis and tissue growth, the Hippo-YAP/TAZ pathway can directly regulate the mTOR pathway by transcriptionally upregulating mTORC1 through an SGK1-dependent mechanism [241]. Moreover, activation of mTOR signaling can enhance YAP/TAZ function by promoting their nuclear localization and stabilization, indicating a direct and reciprocal regulatory connection between the two pathways in metabolic processes [242]. Given the importance of the mTOR pathway in various POL-related traits, this positions the Hippo pathway as a potentially pivotal player in mTOR-related life-history studies.

#### AR signaling

AR signaling supports growth and reproductive function, but excessive or prolonged AR activity has been linked to pro-aging effects, particularly through increased oxidative stress, inflammation, and cancer risk, especially in androgen-sensitive tissues [243–246]. Elevated androgen signaling is also associated with reduced lifespan in male mammals, potentially due to its growth-promoting and mitogenic effects [245–247]. The Hippo–YAP/TAZ pathway can regulate androgen receptor (AR) signaling in multiple ways: at the transcriptional level, it can enhance AR expression, while at the protein level, it can directly inhibit AR through physical interaction [248]. Conversely, AR signaling can promote the dephosphorylation of YAP/TAZ, thereby enhancing their activity [248]. These findings indicate a direct but complex and multilayered regulatory relationship between the two pathways. However, it should be noted that these interactions have been studied primarily in pathological contexts involving excessive cell proliferation, and future studies are essential to validate these mechanisms at the organismal level and in normal life-history contexts.

#### GR signaling

Chronic activation GR signaling, often due to prolonged stress, can lead to immunosuppression, metabolic imbalance, and accelerated aging [249, 250]. High glucocorticoid levels contribute to muscle wasting, insulin resistance, and cognitive decline; features commonly observed in aging organisms [251, 252]. Although GR plays a vital role in acute stress response, its sustained activation undermines longevity-promoting processes. Numerous experimental studies indicate that YAP/TAZ can directly interact with GR signaling pathways in various tissues under both normal and pathological conditions [253–256]. For instance, YAP/TAZ can physically interact with GR at the protein level, inhibiting its activity and modulating GR-responsive gene expression during metabolic processes and under normal physiological conditions [253, 254]. In contrast, GR signaling has also been shown to act as an upstream inducer of YAP/TAZ activity by promoting YAP nuclear localization in both normal and pathological states [255, 256]. These findings highlight a significant point of integration between Hippo signaling and glucocorticoid-mediated effects.

#### TL shortening

Progressive telomere shortening during cell division contributes to cellular senescence and tissue aging, as critically short telomeres trigger DNA damage responses that halt proliferation [214]. This mechanism acts as a molecular clock that limits cellular lifespan and is implicated in age-related degenerative diseases. A key finding in human-derived cells showed that the Hippo co-factor YAP directly regulates *TERT* transcription by binding its promoter, promoting telomerase activity and TL maintenance [257]. Similar YAP-mediated *TERT* regulation has been observed in mice [258], and YAP has also been implicated in *TERC* transcription in cancer studies [259]. In Drosophila, TEAD directly binds telomeric repeats to regulate telomere-specific retrotransposons, affecting TL dynamics [260]. In addition, TAZ, YAP's main partner, modulates TL via two mechanisms: a telomerase-dependent pathway through *POT1*, and a telomerase-independent one via *RAD51 C*; depletion of TAZ reduces expression of both genes, leading to TL shortening [261]. Conversely, TL shortening can enhance YAP activation, while telomerase reactivation suppresses YAP transcriptional activity, as shown in a mouse model [262]. A recent study also suggests reciprocal interactions between YAP/Hippo signaling and telomere regulation in vertebrate gut microbiota–host dynamics [263]. Together, these findings highlight bidirectional links between the Hippo pathway and telomere dynamics, raising the possibility that Hippo's roles in sexual maturation and responses to dietary or thermal changes may be mediated via TL regulation in ecological contexts

### Hippo pathway interactions with POL mechanisms associated with delayed aging and longevity

#### AMPK signaling

AMPK functions as a cellular energy sensor that becomes activated under low-energy conditions, such as fasting or exercise, promoting metabolic adaptations that enhance cellular resilience and survival [128, 264]. By suppressing anabolic processes and stimulating catabolic pathways like autophagy and mitochondrial biogenesis, AMPK helps reduce oxidative damage and improve metabolic efficiency, which are key features of extended lifespan in various model organisms [264, 265]. Unlike the other signals discussed here, the direct regulation of AMPK signaling by components of the Hippo pathway remains to be clarified. However, AMPK’s regulation of the Hippo pathway, such as the inhibition of YAP/TAZ through AMPK-mediated phosphorylation [266], is one of the well-characterized regulatory connections between the Hippo pathway and other signaling mechanisms [266–268]. Given the central role of AMPK in metabolism, this connection may also serve as a key gateway for integrating the Hippo pathway into various metabolic and energy homeostasis processes [269].

#### SIRT1 signaling

SIRT1 plays an important role in promoting longevity by enhancing DNA repair, mitochondrial function, and antioxidant defenses [23, 270]. It exerts its anti-aging effects partly through deacetylating key regulators like FOXO and p53, thereby maintaining genomic stability and stress resistance [271]. SIRT1 also represses inflammation and supports circadian rhythm regulation, both of which deteriorate with age [272, 273]. The Hippo pathway has been shown to directly influence SIRT1 activity through YAP/TAZ, which can enhance SIRT1 transcription [274, 275], and YAP itself is a target of SIRT1-mediated deacetylation [276, 277], establishing a reciprocal regulatory connection between the two pathways. By positioning Hippo–YAP/TAZ among the regulators of SIRT1 signaling, it is not surprising that a range of potential YAP-mediated, SIRT1-dependent mechanisms influencing aging can be envisioned, calling for further investigation.

#### ER signaling

ER signaling has been implicated in lifespan extension, especially in females, through its regulation of metabolic homeostasis, oxidative stress response, and vascular health [243, 278]. ERα, in particular, promotes protective gene expression profiles that enhance mitochondrial function and reduce systemic inflammation [278, 279]. Aging-related decline in estrogen levels is associated with increased disease risk and functional decline [279]. Recent studies in mammalian cells have found that YAP can directly inhibit ERα transcription by binding to an enhancer located upstream of the ERα gene [280, 281]. Interestingly, this inhibitory regulatory connection may be reciprocal, as ER signaling can suppress YAP activity by promoting its phosphorylation [282, 283]. Although these regulatory links appear to be direct, it remains unclear whether their reciprocal inhibition operates under normal physiological conditions, as these findings were all obtained under pathological contexts.

#### TH signaling

Thyroid hormones are essential for maintaining metabolic rate, thermogenesis, and tissue homeostasis, but tightly regulated TH signaling is also associated with extended lifespan in multiple animal models [284, 285]. Mild reductions in TH levels have been linked to lower oxidative stress and improved cellular efficiency, which contribute to delayed aging [284, 286]. TH also plays roles in brain aging and neuroprotection [287]. Experimental evidence demonstrates that Hippo pathway activity, through TAZ and its downstream target PAX8, is essential not only for the development of the thyroid gland but also for thyroid hormone biosynthesis [288, 289]. Although these recent findings indicate an extensive and direct regulatory role of the Hippo pathway upstream of TH signaling, further investigations are required to understand the function of these regulatory connections in ecological and life-history contexts.

#### Leptin signaling

Leptin signaling helps maintain glucose homeostasis, reduces lipid accumulation, and prevents the metabolic decline often seen in age-related disorders [133, 290]. Leptin resistance is commonly associated with obesity and accelerated aging, while leptin sensitivity is linked to metabolic health and longevity [290, 291]. A recent research has shown that Hippo–YAP/TAZ can act as an upstream regulator of leptin signaling by directly binding to an upstream enhancer site of the leptin gene and upregulating its expression in adipocytes [292]. This important finding places Hippo–YAP/TAZ at a key position in leptin-dependent metabolic processes; however, the universality of this regulatory link remains to be explored across taxa and in relation to environmental changes.

#### DLK1-Notch signaling

DLK1-Notch signaling is involved in stem cell quiescence and regenerative capacity, both of which are vital for slowing age-related decline [145, 293]. Proper regulation of this pathway helps maintain the balance between self-renewal and differentiation, preventing premature stem cell exhaustion and tissue degeneration [293]. During adipocyte proliferation, the Hippo pathway directly intersects with the Notch signaling cascade via YAP/TAZ-mediated transcriptional regulation of *DLK1*, indicating a clear upstream influence of Hippo components on DLK1–Notch signaling activity [294]. Inhibition of YAP/TAZ function by LATS2 leads to reduced *DLK1* transcription, subsequently blocking the inhibitory effects of *DLK1* on adipogenesis [294]. This further suggests that the Hippo–YAP/TAZ regulatory axis may play an essential role in DLK1–Notch-dependent regulation of energy balance and metabolism; an area that requires further investigation in ecological contexts.

### Examples of Hippo pathway interactions with other mechanisms influencing aging

#### DNA methylation

DNA methylation is a key epigenetic mechanism that regulates gene expression and genome stability, and its role in aging is highly context-dependent, even though aging is characterized by a global decrease in methylation alongside site-specific increases at particular genomic loci [295, 296]. Age-related changes in DNA methylation can reflect both protective adaptations and harmful deregulation [297, 298]. While hypermethylation of tumor suppressor genes or hypomethylation of repetitive elements can accelerate genomic instability and aging, targeted methylation changes are also involved in longevity-associated gene regulation [297]. DNA methylation patterns form the basis of epigenetic clocks, which closely track biological aging [299]. Components of the Hippo pathway, particularly YAP/TAZ, are known to influence DNA methyltransferase activity and chromatin accessibility, thereby shaping the epigenetic landscape in a way that can impact both development and aging trajectories. For instance, some key players in the DNA methylation process, namely DNMT1, TET1, and EZH2, are known to be direct targets or interacting partners of the Hippo–YAP/TAZ pathway [300–302], while DNMT1 and EZH2 can also act directly upstream of YAP/TAZ signaling [303–305]. Interestingly, these same DNA methylation factors have also been implicated in regulating the onset of sexual maturation [306, 307] and longevity [308]. These complex, reciprocal regulatory connections offer a myriad of possibilities across biological processes involving both DNA methylation and the Hippo pathway.

#### RNA methylation

RNA methylation, particularly N6-methyladenosine (m6 A) modification, is a rapidly emerging regulator of gene expression, RNA stability, and translation efficiency, all of which have context-dependent impacts on aging [309, 310]. Depending on the cellular environment and which m6 A writers (e.g., METTL3), erasers (e.g., FTO), or readers (e.g., YTHDF proteins) are active, m6 A can either promote longevity by enhancing stress responses and repair mechanisms or accelerate aging through increased inflammation and impaired differentiation [309, 310]. Importantly, recent evidence suggests that m6 A methylation is responsive to numerous environmental cues, including nutrient levels, oxidative stress, and temperature fluctuations, positioning it as a molecular sensor linking environmental conditions to gene regulation [311]. The Hippo pathway intersects with this process, as YAP/TAZ activity can be directly influenced by m6 A methylation process (e.g. via FTO and METTL3) [312, 313], establishing important connections that can integrate the Hippo pathway to environmental sensing and aging through epitranscriptomic.

#### MicroRNAs

MicroRNAs (miRNAs) fine-tune gene expression post-transcriptionally and play complex roles in aging, acting as either accelerators or suppressors, depending on the specific miRNA, tissue context, and target pathways [314, 315]. For instance, some miRNAs promote senescence, inflammation, or DNA damage, while others support stem cell maintenance, stress resistance, and metabolic balance, contributing to extended healthspan [314, 315]. The Hippo pathway is both a target and regulator of miRNAs: YAP/TAZ are directly repressed by several aging-associated miRNAs [316–318], while YAP itself can regulate miRNA processing enzymes like Dicer and Drosha [85, 316], establishing a feedback loop that links Hippo signaling to the miRNA network in aging. Moreover, it is interesting to note that the Hippo pathway can also act directly as an upstream regulator of specific microRNAs, such as *miR- 29* and *let- 7* [85, 319], which are well known for their roles in aging processes and the modulation of longevity [320, 321] as well as pubertal timing [86, 322].

## Unanswered questions and future directions

We have examined how a diverse set of molecular mechanisms, including hormonal signaling, metabolic sensing, and epigenetic regulation, contribute to the regulation of POL traits, with reproductive timing serving as a key example. Emerging evidence places the Hippo pathway at the center of these regulatory networks, acting both upstream and downstream of many of these signals. Rather than operating in isolation, these mechanisms interact dynamically, often responding to environmental cues such as energy availability, temperature, and stress exposure. The Hippo pathway, with its capacity to integrate and coordinate these inputs, presents a compelling candidate for understanding how organisms modulate POL strategies across ecological contexts. Yet, many questions remain about how these interactions function at the organismal level and vary across taxa—offering fertile ground for future research.

### From pathological models to organismal and ecological relevance

A recurring theme across the studies discussed in this review is that many of the direct regulatory connections between the Hippo pathway and POL-related mechanisms have been identified primarily in mammalian cell lines or under pathological conditions, such as cancer or tissue overgrowth. These models, while informative for uncovering the basic molecular interactions, do not always reflect the normal physiological states in which POL traits evolve and operate. This context presents an important limitation. The relevance of Hippo–POL interactions under normal biological conditions, particularly those involving growth, metabolism, and reproductive timing, remains largely untested. Understanding whether these regulatory links function similarly in non-pathological settings is essential for assessing their significance in life-history evolution. Furthermore, most current data come from a narrow range of model organisms, particularly mammals, limiting the ability to generalize findings across taxa with diverse POL strategies. To address these gaps, future experimental studies should prioritize:

1. Validating known Hippo–POL interactions under normal physiological conditions, including during development, growth, and reproductive transitions.
2. Shifting the focus from cell-based models to whole-organism studies, where POL traits manifest in integrated, multi-systemic ways.
3. Expanding research beyond mammals to include species with contrasting ecological life-history strategies, which may reveal conserved or divergent roles of the Hippo pathway.
4. Investigating how environmental variables, such as food availability, temperature, and stress, modulate Hippo signaling and its downstream targets in ecologically realistic contexts.

Bridging the current gap between molecular findings and ecological function will be key to understanding the true scope of the Hippo pathway's role in shaping POL traits. Such research could uncover how this evolutionarily conserved signaling network contributes to the adaptive tuning of life-history strategies across species and environments.

### The Hippo pathway as a regulator of aging and longevity: Moving beyond growth and maturation

Research on the Hippo signaling pathway has primarily focused on its central roles in regulating body growth and the timing of reproductive maturation. These developmental endpoints have offered important insights into how Hippo signaling integrates environmental and physiological signals to shape life-history trajectories. However, this narrow focus has left a substantial gap in our understanding of the Hippo pathway's broader role in other POL-related traits, particularly those associated with aging and longevity. The Hippo pathway interacts with a wide range of molecular mechanisms that are known to influence lifespan and the rate of aging. Components such as YAP and TAZ are tightly connected to longevity-regulating signals, including AMPK, mTOR, SIRT1, GR, and key epigenetic modifiers. These interactions position the Hippo pathway as a potential integrator of metabolic regulation, stress responses, DNA repair, and somatic maintenance, processes that are fundamental to aging biology. Despite these strong mechanistic links, the involvement of the Hippo pathway in aging and lifespan regulation remains largely unexamined in both experimental and eco-evolutionary research. Incorporating aging-related traits into Hippo pathway research would significantly broaden our understanding of how organisms manage life-history trade-offs over time. Investigating how Hippo signaling influences the allocation of energy toward repair and maintenance in later life could help explain variation in aging rates across species with different POL strategies. Furthermore, examining its role in late-life decline, regeneration, and longevity under natural conditions may uncover species-specific patterns of Hippo activity that are currently hidden by the reliance on early-life developmental models. Bringing POL traits such as aging and longevity into the scope of Hippo-related research would also help bridge biomedical findings with ecological and evolutionary perspectives. Much of what we know about the Hippo pathway comes from studies on disease and tissue overgrowth, yet its evolutionary functions likely extend to maintaining organismal integrity over the lifespan. Shifting the research lens beyond growth and reproduction would allow for a more comprehensive understanding of the Hippo pathway's role in coordinating life-history strategies in response to environmental pressures.

### Investigating the role of the Hippo pathway in TL changes

While pathways such as mTOR, IGF, and GR signaling have been extensively studied in relation to the Hippo pathway, its role in the regulation of telomere dynamics remains comparatively underexplored compared to many of the mechanisms discussed in this review. Given its established roles in cell proliferation, growth regulation, and emerging evidence of involvement in telomere maintenance, Hippo signaling is a promising candidate for future investigations into TL dynamics in eco-evolutionary contexts. Its influence on stress responses, energy homeostasis, and developmental transitions suggests it may mediate TL-associated trade-offs in life-history evolution. Direct links between Hippo components and telomere machinery support its potential as a molecular bridge connecting environmental variation, TL regulation, and reproductive timing. Species-specific variation in these interactions may further clarify Hippo’s evolutionary significance. Moreover, while many TL-associated genetic loci have been identified, their functional regulation remains poorly understood [208]. One notable example is E2 F1, a conserved transcription factor involved in organ size [323], testicular maturation [324], and telomerase expression [325], which also inhibits YAP activity [323], highlighting a potential integrative link between Hippo signaling, TL regulation, and pubertal timing (Table 1).

**Table 1:** Examples of Hippo pathway or telomere length links to thermal and dietary changes at organismal level

**Pathway** **/Mechanism**	**Link to environmental factor**	**Species**	**References**
Hippo pathway (via Lin28)	Diet-induced precocious puberty	Mice (*Mus musculus*)	[80, 81]
Hippo pathway (via Yap)	Diet-induced obesity	Mice (*Mus musculus*) Human (*Homo sapiens*)	[68] [69]
Hippo pathway	Adaption to low temperature	Honey bees *(Apis mellifera)*	[104]
Hippo pathway	Adaption to different temperature gradients	Hive beetles *(Aethina tumidahas)*	[102]
Hippo pathway	Adaption to thermal stress	Oysters (*Crassostrea spp.)*	[106]
Hippo pathway	Adaption to high temperature	Indigenous chickens (*Gallus gallus spadiceus*)	[107]
Hippo pathway	Adaption to low temperature	Giant pandas (*Ailuropoda melanoleuca*) Pig (*Sus scrofa*)	[109] [108]
Telomere length	Warmer temperature	American alligators (*Alligator mississippiensis*) Desert lizard (*Phrynocephalus przewalskii*)	[326]
Telomere length	Environmental temperature in association with age at maturity	Nine-spined sticklebacks (*Pungitius pungitius*)	[220]
Telomere length	Environmental temperature depending on life stage	Zebra finch (*Taeniopygia castanotis*)	[327]
Telomere length	Environmental temperature depending on the tissues	Human (*Humo sapien*)	[328] [329]
Telomere length	Dietary fat composition	Mice (*Mus musculus*)	[226] [330]
Telomere length	Food availability	Atlantic salmon (*Salmo salar*)	[229]
Telomere length	Dietary fat composition	Great tit (*Parus major*)	[230]
Telomere length	Food availability	Western spadefoot toad (*Pelobates cultripes*)	[331]

### Need for balanced research expansion

Although increasing evidence points to the Hippo pathway as a key integrator of molecular mechanisms underlying POL traits, many of its proposed connections, such as those with metabolic regulators, hormonal signals, epigenetic modifiers, and telomere-associated processes, remain unevenly studied. A notable limitation in the current literature is the lack of systematic validation across diverse biological models, life stages, and environmental contexts. While some links have been supported in mammalian systems, their generality and ecological relevance are still uncertain. To address this gap, future studies should move beyond correlative findings and adopt experimental designs that can establish causality. This includes the use of loss-of-function and gain-of-function models for Hippo components in relation to various POL-associated pathways. Incorporating non-mammalian species and ecologically relevant conditions will be essential to determine whether observed regulatory interactions are conserved or context dependent. Another important step is ensuring methodological and taxonomic diversity, as well as the inclusion of negative findings. The lack of published null results may contribute to an inflated sense of functional connectivity, hindering the refinement of mechanistic models. Encouraging the dissemination of such results will help reduce publication bias and support a more balanced perspective. By expanding the scope of research and applying rigorous, context-aware approaches, we can build a more accurate understanding of how the Hippo pathway interfaces with the molecular systems that govern POL traits. This will ultimately strengthen its integration into eco-evolutionary frameworks and help clarify its role across species and environments.

## Data Availability

Not applicable.
